# Extent of wall motion abnormalities with cardiovascular magnetic resonance imaging overestimates the degree of coronary artery disease in patients with ischemic cardiomyopathy

**DOI:** 10.1186/1532-429X-13-S1-P125

**Published:** 2011-02-02

**Authors:** Tatyana Danilov, Andrea Mignatti, Ruth P Lim, Leon Axel, Monvadi B Srichai

**Affiliations:** 1 New York Univeristy Medical Center, New York, NY, USA

## Background

Patients with ischemic cardiomyopathy (CMP) often develop regional wall motion abnormalities (WMA) due to myocardial scarring, stunning or hibernation. Abnormal functioning myocardial segments without evidence of fibrosis on late gadolinium-enhancement cardiac magnetic resonance (LGE-CMR) are felt to represent stunned or hibernating myocardium, presumably with significant coronary artery disease (CAD) in the vessel supplying the region. We analyzed the association between segmental wall motion abnormalities (WMA) and underlying CAD in ischemic CMP.

## Methods

53 consecutive patients referred for LGE-CMR with recent coronary angiography (< 2 weeks from index CMR) and evidence of ischemic CMP on LGE-CMR (subendocardial or transmural LGE) were included. CMR was analyzed using AHA 17-segment model for regional function and late gadolinium enhancement (LGE) as well as global left ventricular (LV) size and function. Myocardial segments were assigned to angiographic coronary artery territories based on AHA standardized segmentation for correlation of CMR and angiographic data.

## Results

Of 901 segments, 638 (71%) had WMA, with severe WMA in 378 (42%). In contrast, only 340 (38%) had LGE, including 233 (24%) with >50% transmural LGE, consistent with larger extent of WMA compared to LGE. No significant correlations were found between segments with any WMA and >50% transmural LGE (r= 0.05, p=0.71.). Of the 53 patients with ischemic CMP by LGE-CMR, 47 patients (89%) had documented coronary artery disease by angiography. There was no significant correlation between number of vessels with severe stenoses (>70%) and any WMA (r=0.19, p=0.18). Additionally, the number of segments with evidence of any WMA was more extensive than number of segments supplied by severe stenoses.

## Conclusions

Patients with ischemic CMP demonstrate extensive LV WMA out of proportion to the extent of myocardial scarring and epicardial CAD. These findings suggest that additional mechanisms such as microvascular disease, LV remodeling, inflammation or hibernation extending into other myocardial segments not supplied by a critical stenosis may contribute to the degree of WMA in patients with ischemic CMP.

